# Painting the Lily

**Published:** 1875-01

**Authors:** 


### PAINTING THE LILY.

To paint the lily and adorn the rose do not appear to be impossiblities, after all. An Italian Professor, L. Gabba, claims to have accomplished a good deal in this direction, and communicates the result of his experiments to the *Journal* of the French Central Society of Horticulture. He changes the color of various flowers at will, using aqua ammonia as the agent. His first process was to pour a little ammonia water into a plate and invert a funnel over it and then insert the flower in the tube of the funnel. In this way blue, violet, and purple flowers become of a fine green color; deep carmine-colored flowers, such as pinks, become black; white blossoms turn yellow, and so on; but the most curious effects are produced on variegated flowers, such as red and white, when the former is changed to green and the latter to yellow. Fuchsias, with white and red flowers become yellow, blue, and green by exposure to the ammoniated fumes. When the colors have been thus changed, if the blossom be dipped in pure water it will retain the artificial color for some hours, and will afterward return gradually to its natural tint. Perhaps the most curious observation in this connection is that the Aster, which is naturally without scent, actually acquires an aromatic and agreeable odor under the influence of ammonia. Experiments tried with acid solutions also produced singular results. Thus, flowers of a violet color become red when sprinkled with water containing nitric acid; and if inclosed in a wooden box and exposed to the action of the fumes of muriatic acid, known as hydrochloric acid gas, will in six hours become of a fine carmine color, which they will retain if first dried in a dark place and kept dry in the shade.

Swallowing a Cent.—Dr. Gibbs, one of the editors of this Journal, who is himself an educated physician and surgeon, while on a railroad train, the other day, was consulted by one of the employes on the cars in relation to his little boy who had that morning swallowed a cent.

"What have you done for him," asked the Doctor. "We gave him a dose of castor oil," was the reply. "Good practice, so far; as soon as you reach home give him the whites of three raw eggs daily, let his diet be bread and milk, and *nothing sour.*" The directions were followed faithfully, the whites of the eggs repeated every day, and the dose of oil at night, and on the fourth day the cent was discharged. It was one of the new copper coins, and considerably coroded by the action of the gastric juices. Since fatal results often follow the swallowing of copper coin, the judicious treatment advised in this instance should be remembered by all who have the care of children. The essential points to be borne in mind are simply these: albumen, or the whites of eggs, a bland diet free from acids, and castor oil.

---

All who are disgusted with disease and anxious to be rid of their ails, will read with interest, between their doses, the story of Jacob Weiss, who formerly lived, a mechanic, in Louisville, Ky., with a plenty of nothing in his pocket, and bronchitis slowly but surely consuming his pulmonary apparatus. He was advised to go to that Paradise, Golconda, Arcadia, that argentine land called Colorado. What happened to the impecunious and with difficulty-breathing Jacob? He recovered from his bronchial derangement—but that is nothing. For he also discovered six large and plentiful silver mines. He is a rich man. If necessary, he could afford to take a bottle of the most expensive Pulmonic Panacea twice a day, and to employ a corps of doctors, allopathic, hydropathic, motorpathic, homeopathic, botanical and eclectical. But he scorns them. He breathes with the vigor of a bison? Consumption has stepped out—capital has stepped in. Let those whose tendency is to shortness both of funds and breath, go and do like-Weiss !

